# Editorial: Enhancing salinity tolerance in crop plants through agronomic, genetic, molecular, and physiological approaches

**DOI:** 10.3389/fpls.2025.1554509

**Published:** 2025-03-11

**Authors:** Muhammad Umair Hassan, Muhammad Nawaz, Lorenzo Barbanti, Sajid Masood

**Affiliations:** ^1^ Research Center on Ecological Sciences, Jiangxi Agricultural University, Nanchang, China; ^2^ Department of Agricultural Engineering, Khwaja Fareed University of Engineering and Information Technology, Rahim Yar Khan, Pakistan; ^3^ Department of Agricultural and Food Sciences, University of Bologna, Bologna, Italy; ^4^ Department of Soil Science, Faculty of Agricultural Sciences and Technology, Bahauddin Zakariya University, Multan, Pakistan

**Keywords:** salinity, resistance mechanism, abiotic stress, biotechnological approaches, sodium

Soil salinity is increasing worldwide, posing a serious threat to crop production and food security. The rate of increase in salinity is estimated at 1.5 million hectares per year, which is a major challenge in the context of rising food demand. Ongoing climate change is contributing to exacerbating the effects of salinity through erratic precipitations that fail to leach soluble salts below the rhizosphere. We still need to cope with a wider diffusion of salinity and its related effects on both natural and cultivated land. Plant tolerance mechanisms, the ways to harness them and the complementary crop management practices constitute the comprehensive domain in which responses to this adversity should be sought. The authors who participated in this Research Topic have contributed to this quest for solutions.

Both large-scale crops, namely wheat and maize, and target species such as *Aegilops tauschii* were involved in the attempts to shed light on stress resistance mechanisms, their genetic expression, and external means to enhance them. On one side, molecular, biochemical, omic and other biotechnological and genetic approaches were investigated. On the other side, exogenous supplementation of elements (Si and Se), amendments (biochar), plant growth regulators and beneficial microorganisms were tested. However, biotechnological/genetic approaches were the main focus of the majority of the works in this Research Topic. Additionally, several works focusing on external inputs relied on biotechnological applications to better understand the reasons for their effects.

Particular attention was paid to Na^+^ plant entry and translocation between organs, and its relationship with other cations, namely K^+^. In transgenic *Brassica rapa*, excess Na^+^ was removed from the transpiration stream by means of high-affinity K^+^ transporter proteins (Irulappan et al.). This resulted in higher biomass and longer roots under salt stress than in non-transgenic plants. A bread wheat (*Triticum aestivum*) genotype cloned with a gene from *Arabidopsis thaliana* that was found to be upregulated under salinity stress showed better growth under salinity stress and demonstrated a higher ability to retain K^+^, suggesting that the specific gene acts as an elicitor to sequester Na^+^ ions in cell vacuoles while retaining more cellular K^+^ (Imtiaz et al.). Even in commercial bread wheat cultivars compared to synthetic hexaploid genotypes, specific overexpressed genes found only in the latter genotypes determined a significant decrease in the Na^+^/K^+^ ratio under salt stress (Alghabari and Shah). These genotypes are, therefore, seen as a potentially useful source of genes for superior wheat cultivars.

The relationship of sodium with other cations was also the focus of a work addressing Si supplementation as Ca_2_SiO_4_ in a salt-sensitive vs. tolerant maize variety under salt stress (Mahmood et al.). Silicon supplementation increased maize growth, intracellular ion (including K^+^) homeostasis and reduced Na^+^ concentration in leaf apoplasts, suggesting the use of Ca_2_SiO_4_ as an agent for salinity mitigation. Similarly, selenium supplementation to tomato (*Solanum lycopersicon*) seedlings was shown to hinder Na^+^ root uptake and translocation to leaves, rebuilding ionic homeostasis by increasing the K/Na, Ca/Na and Mg/Na ratios, while fostering higher photosynthetic capacity (Zhang et al.).

Biochar is equally deemed useful in mitigating the adverse impact of soil salinity, as it improves nutrient homeostasis, reduces the accumulation of toxic ions such as Na^+^ and Cl^-^, and ameliorates a number of plant physiological processes (Huang et al.). Similarly, the contribution of biochar to soil properties such as soil structure, aggregate stability and water-holding capacity may not go unnoticed under conditions of soil salinity (Wu et al.). Among exogenous plant inputs, the application of a growth regulator as valine-threonine-isoleucine-aspartic acid to maize (*Zea mays*) seedlings under salt stress remarkably ameliorated plant growth and enhanced the activity of antioxidant enzymes and the level of osmoregulatory substances, while reducing the contents of oxidative stress markers and determining changes in the metabolomic profile of lipids and lipid-like molecules (Wu et al.). Soil application of five types of beneficial microorganisms to hot pepper (*Capsicum annuum*) grown on saline soil attenuated the salt-induced damage to plant growth and yield, increased capsaicin content and increased the parameters of the antioxidant defense system (Abdelkhalik et al.). Finally, the partnership of humic acid and potassium increases salt tolerance by improving plant function and soil properties (Ahmad et al.).

Plants have developed different morphological and molecular strategies to withstand salinity stress. Testing genetic variability in diverse gene pools and subsequently developing salt-tolerant crops has been shown to be an effective way to counter salinity stress for sustainable crop production (Atta et al.). Cui et al. identified 59 *SbPUB* genes from the sorghum genome, and they clustered these genes into five groups based on their conserved motifs and structures. The proteomic and transcriptomic analysis revealed that many *SbPUB* genes had variable expression under salt stress. Therefore, they could be an important target for breeding salt-tolerant sorghum genotypes.

Salinity and drought stress substantially affect the growth and biomass production of *Aegilops tauschii*. However, the physiological traits of this plant improved with increasing nitrogen availability and plant density. These two conditions also increased the expression of plant genes that play a remarkable role in countering drought and salinity stress (Hameed et al.).

Huang et al. documented that rapid alkalinization factors (RALFs) are widely present in plants, and they induce cell expansion by interacting with inter- and intracellular deposition of cell wall (CW) materials, and by rearranging the materials already present in the CW. Melatonin is an important growth hormone, whose application induced physiological, molecular and anatomical changes in switchgrass plants by increasing the expression of hydroxylindole O-methyltransferase (*oHIOMT*) in a dose-dependent manner (Huang et al.). A lower concentration of melatonin improved *oHIOMT*, while a high concentration inhibited *oHIOMT* expression and subsequent plant function (Huang et al., 2024). A salt-tolerant gene (*OsBBX11*) was identified in *japonica* rice and found to play a crucial role in salt tolerance by modulating the transport of Na^+^ and K^+^ from the soil to the aerial parts of the plant (Lei et al.). Rasool et al. identified five pairs of syntenic GrTCS homologs in the *Gossypium raimondii* genome. They found that the GrTCS genes were differentially regulated in response to cold and salt stress, indicating a potential role for GrTCS in salt tolerance mechanisms.

In conclusion, from this overview it appears that in salinity mitigation, historically based on hydrological countermeasures and levels of plant resistance, current contributions have mainly turned their attention to biotechnological tools in search of mechanisms of stress resistance. However, no univocal tool/process has been addressed. This makes the results varied, paradigmatic, and very stimulating in terms of downstream applications. In other words, the door is open for biotechnological/genetic applications to improve plant resistance to salinity stress, but any given application must be tailored to each specific plant/environmental condition.

